# Supplementary material for: Human Complement Inhibits Myophages against Pseudomonas aeruginosa
Source: Viruses. 2023 Nov 3;15(11):2211. doi: 10.3390/v15112211 (PMC10674969; doi:10.3390/v15112211)
Supplement: Supplementary file 1 [file viruses-15-02211-s001.zip › viruses-2665479-supplementary.pdf]

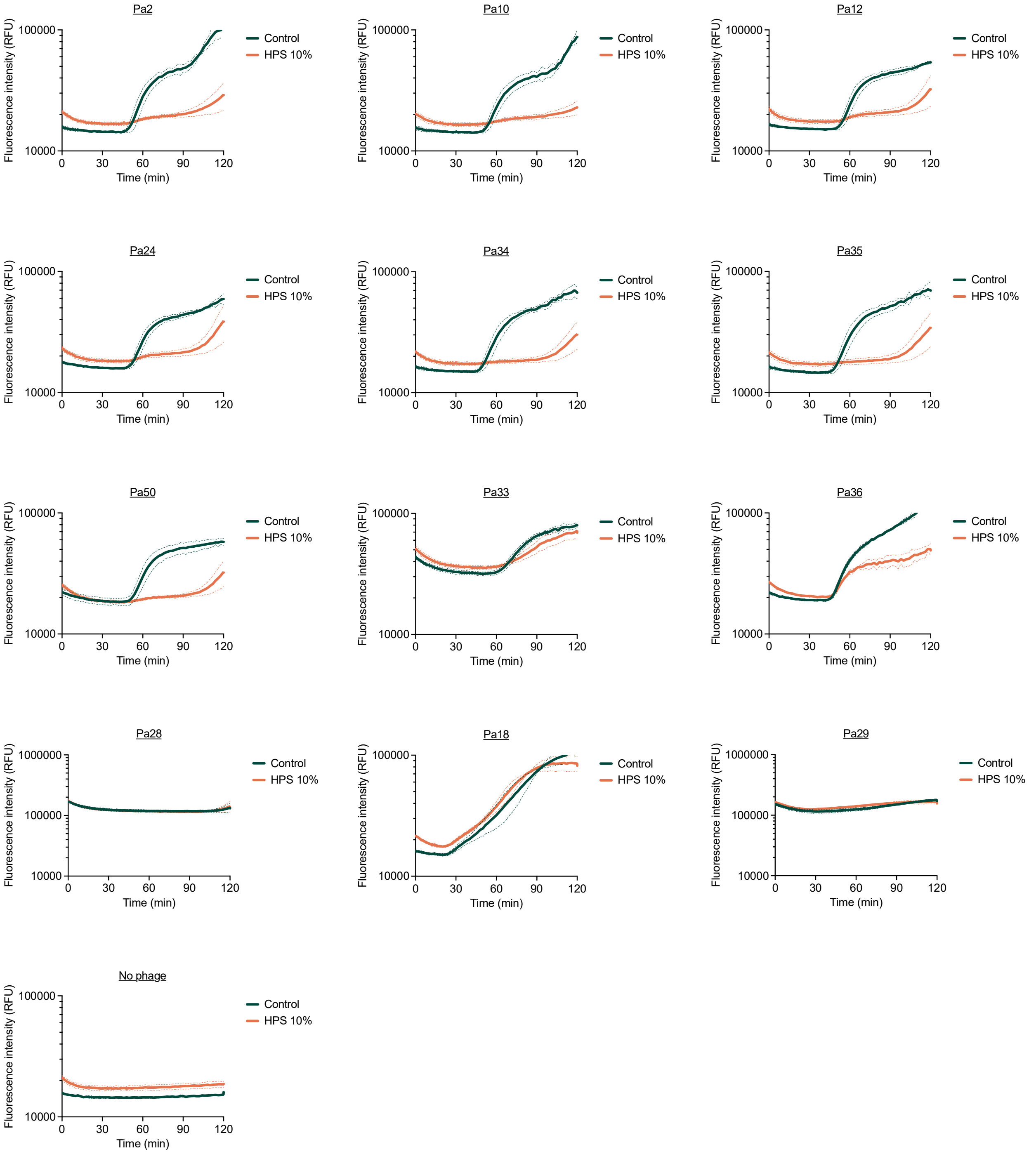

Figure S1. Human serum inhibits a variety of myophages targeting PAO1. Fluorescence intensity (relative fluorescence units, RFU) over time of PAO1 infected at 37°C in presence of the DNA dye Sytox green with different phages (MOI 1) in absence of serum (control) of in 10% HPS. Data represent mean ± SD of three independent experiments.

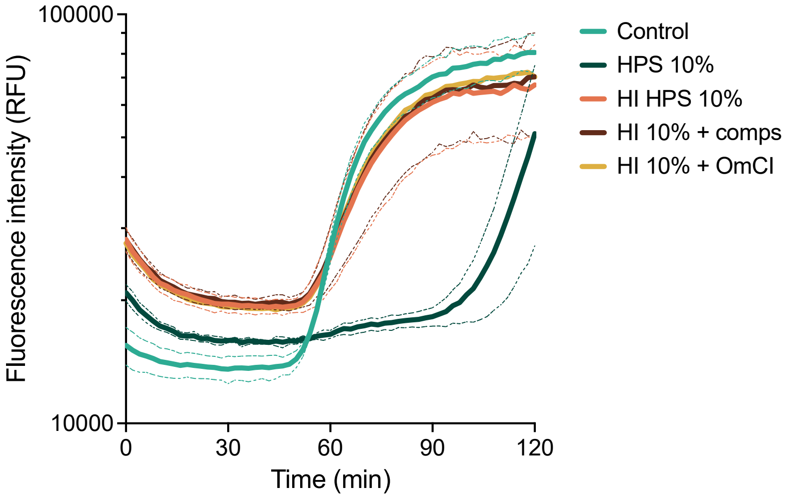

Figure S2. Inhibition of phages by serum is mediated by the complement system. PAO1 was incubated with phage PBJ at 37ºC in presence of the DNA dye Sytox green. Data represents fluorescence intensity (RFU) over time (min) of bacteria infected with PBJ at an MOI of 10 (control), in presence of 10% HPS, 10% HI HPS, 10% HI HPS with 50 μM compstatin, or 10% HI HPS with 20 μg/mL OmCI. Data represent mean ± SD of three independent experiments.

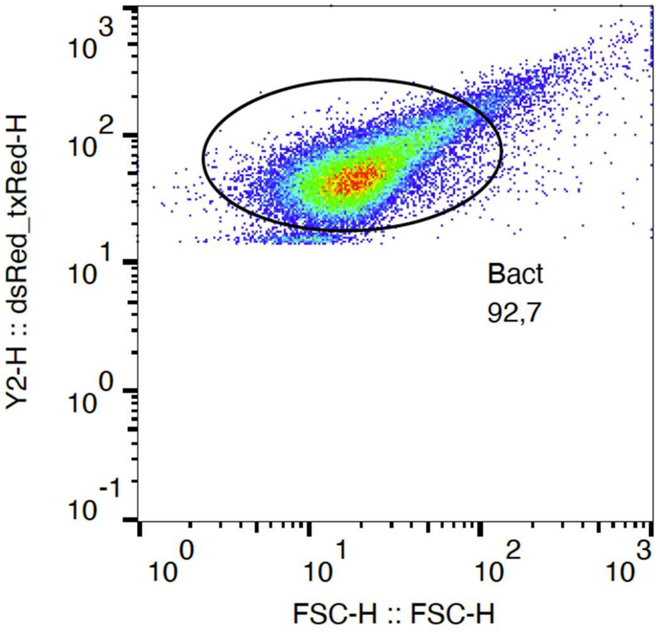

Figure S3. Example of gating strategy of PAO1-sfCherry for flow cytometry experiments. Y axis shows high of sfCherry signal, X axis shows height of forward scatter signal. Sample shown in this case are bacteria treated with 10% HPS for 5 minutes at 37ºC.
